# A Repertory of Hering’s Guiding Symptoms

**Published:** 1896-01

**Authors:** 


A REPERTORY OF HERING'S GUIDING SYMPTOMS OF OUR MATERIA MEDICA. By Calvin B. Knerr, M. D., Philadelphia. Published by The F. A. Davis Co., for the Estate of Constantine Hering, 1896. 1232 pages; sheep, $10.00.

It is with much pleasure that the editor of THE HOMŒOPATHIC PHYSICIAN announces to the profession the completion of the long-desired Index or Repertory to *The Guiding Symptoms.* To the earnest seeker after the simillimum, we cordially recommend this magnificent work with the assurance that in its pages he will meet no disappointment; for if the symptom he wishes to find is in *The Guiding Symptoms* at all, he will certainly be able to locate it by an attentive study of this excellent book, and that, too, in a reasonably short time. Those of us who have spent hours and hours ransacking the *Materia Medica* for some symptom urgently needed to select a remedy to cure a case, will know the value of this book. Even the careless generalizing prescriber, who sneers at the pains-taking "symptom coverer," and refuses to emulate his example of patience and perseverance, will find in this book an incentive to a closer study of the *Materia Medica*, and gradually and perhaps almost unconsciously, to become a better homœopathist.

Those familiar with *The Guiding Symptoms*, will remember that the pathogenesis of each remedy is divided into sections, called by Dr. Hering "chapters." These chapters are numbered and provided with titles or headings in antique black letter.

The number always corresponds to the title of the chapter, no matter under what remedy it occurs. Thus "Inner Head" is always number three. "Throat" is number thirteen. "Cough and Expectoration" is number twenty-seven, and so on, no matter where these chapters occur, and whether the pathogenesis be furnished with the full number of chapters or whether it be abbreviated.

In the plan of the *Repertory of Hering's Guiding Symptoms*, now under review, the work is divided similarly into chapters having the same headings and the same numbers without exception. Any given chapter in the Repertory contains, therefore, all the symptoms to be found in the same chapter in every proving given in the whole ten volumes of *The Guiding Symptoms.*

A glance at the top of the chapter will show every one of the greater sub-headings to be found in that chapter. These sub-headings are in black letter, the better to catch the eye, and are in alphabetical order. For example, let us take the chapter " Inner Chest and Lungs," which we find under any of the remedies in *The Guiding Symptoms* with the number twenty-eight. At the head of this chapter in the Repertory we find a catalogue of sub-headings. They are as follow: "Clavicles, Inner Chest, Lungs, Sternum." Running the eye down the page we find " Inner Chest " in large black letters. Then under this sub-heading, in smaller black letter, we find paragraphs in alphabetical order, *abdomen, abscess, aching, affections* (in general), *alive, angina pectoris, anxiety, back, ball, bladder, blows, boring, brain, breathing*, etc.

Looking now at the paragraph labeled *abdomen* for example, we find the following symptoms: " Bowel symptoms seemed to alternate with inner chest (dropsy), Dig." "Pains alternate, Æscul-h." "Pains extend to abdomen (epidemic affection), Card-m."

Let us take another paragraph, " angina pectoris." Here sixty-one remedies are mentioned, followed by a long list of particular differential indications such as, " sudden anxiety, Tabac ;" " loss of appetite, and gas in the bowels, Nux-v. ;" " while climbing, Coca ;" " from excitement and exertion with slow pulse, Cup-m. ;" " during attack fearing some organic lesion of the heart will cause sudden death, Cactus," and so on. At the end of the paragraph we find a pointing hand with cross references, thus: ☞ Anxiety, congestion, constriction, fullness, neuralgia, oppression, pressing. These cross references are very numerous and are one of the most distinguishing characteristics of the book. They serve to direct the student to every place where information and indications may be found upon any given ailment, and that, too, with a minimum expenditure of time.

In the editorial in the December number of this journal, at page 539, a number of symptoms of starting will be found. Let us look at this symptom in the Repertory now under review. How shall we find it? It may be under " Nerves," chapter 36, or it may be under " Sleep," chapter 37. We will look at the index—for this complete repertory is even furnished with an alphabetical index—and see if we can find that word there. Under the letter S, we find it, and we are referred to page 1,034 in chapter 36, Nerves. Here we find, starting under every possible condition except one—sleep. At this latter word we find a cross reference, thus : " ☞ Chap. 37, during sleep starting."

We now turn to chapter thirty-seven—which is an easy thing to do, for the name of every chapter and its number are repeated conspicuously at the top of each page—and run the eye down the paragraph sub-headings. These, as before stated, are in alphabetical order. We arrive at the word " starting," and there find an abundant list of indications of starting when on the point of falling to sleep, given with such particular detail as to render utterly insignificant the list in the editorial before mentioned.

This paragraph is followed by two cross references to "starting on awaking" and "twitching." Thus everything pertaining to this subject is brought under the eye of the physician, and, as before stated, in a very short time.

We think enough samples are here furnished to give the reader an idea of the great value of the book, the elaborate and yet comprehensive plan upon which it is built, and its general merit in rendering almost absolutely certain the finding of any symptom, if it be in *The Guiding Symptoms*, and of saving much time in getting it. We have abundantly proved that we can, in five minutes, find a symptom which formerly would take five hours of close searching.

The editor can say for himself that the book had not been in his possession ten minutes before he had used it in making a prescription, and before half an hour had passed he had made two good prescriptions from it. We cordially recommend it to all. If the cost seems formidable we can only say that any such consideration as that should not stand in the way. Make any sacrifice to obtain a copy, secure in the conviction that the investment will richly repay you in the end.

